# Persulfidation of plant and bacteroid proteins is involved in legume nodule development and senescence

**DOI:** 10.1093/jxb/erad436

**Published:** 2023-11-11

**Authors:** Manuel A Matamoros, Luis C Romero, Tao Tian, Ángela Román, Deqiang Duanmu, Manuel Becana

**Affiliations:** Departamento de Biología Vegetal, Estación Experimental de Aula Dei, Consejo Superior de Investigaciones Científicas, Avenida Montañana 1005, 50059 Zaragoza, Spain; Instituto de Bioquímica Vegetal y Fotosíntesis, Consejo Superior de Investigaciones Científicas y Universidad de Sevilla, 41092 Sevilla, Spain; National Key Laboratory of Agricultural Microbiology, College of Life Science and Technology, Huazhong Agricultural University, Wuhan 430070, China; Departamento de Biología Vegetal, Estación Experimental de Aula Dei, Consejo Superior de Investigaciones Científicas, Avenida Montañana 1005, 50059 Zaragoza, Spain; National Key Laboratory of Agricultural Microbiology, College of Life Science and Technology, Huazhong Agricultural University, Wuhan 430070, China; Departamento de Biología Vegetal, Estación Experimental de Aula Dei, Consejo Superior de Investigaciones Científicas, Avenida Montañana 1005, 50059 Zaragoza, Spain; University of Warwick, UK

**Keywords:** Hydrogen sulfide, legumes, nodule senescence, persulfidation, post-translational modifications, symbiotic nitrogen fixation

## Abstract

Legumes establish symbiosis with rhizobia, forming nitrogen-fixing nodules. The central role of reactive oxygen species (ROS) and reactive nitrogen species (RNS) in nodule biology has been clearly established. Recently, hydrogen sulfide (H_2_S) and other reactive sulfur species (RSS) have emerged as novel signaling molecules in animals and plants. A major mechanism by which ROS, RNS, and RSS fulfil their signaling role is the post-translational modification of proteins. To identify possible functions of H_2_S in nodule development and senescence, we used the tag-switch method to quantify changes in the persulfidation profile of common bean (*Phaseolus vulgaris*) nodules at different developmental stages. Proteomic analyses indicate that persulfidation plays a regulatory role in plant and bacteroid metabolism and senescence. The effect of a H_2_S donor on nodule functioning and on several proteins involved in ROS and RNS homeostasis was also investigated. Our results using recombinant proteins and nodulated plants support a crosstalk among H_2_S, ROS, and RNS, a protective function of persulfidation on redox-sensitive enzymes, and a beneficial effect of H_2_S on symbiotic nitrogen fixation. We conclude that the general decrease of persulfidation levels observed in plant proteins of aging nodules is one of the mechanisms that disrupt redox homeostasis leading to senescence.

## Teaser

Hydrogen sulfide and persulfidation are important in redox signaling during bean root nodule development and senescence.

## Introduction

Reactive oxygen species (ROS) and reactive nitrogen species (RNS) perform essential functions in plant cells. Thus, superoxide radicals, hydrogen peroxide (H_2_O_2_), and nitric oxide (NO) accumulate transiently at specific cellular sites and trigger signaling cascades. However, their concentrations need to be tightly regulated by antioxidant enzymes and metabolites to maintain intracellular redox homeostasis (for recent bibliography see Umbreen *et al.*, 2018; Castro *et al.*, 2021; Minguillón *et al.*, 2022). In legume nodules, different isoforms of superoxide dismutases (SODs) catalyse the dismutation of superoxide radicals to H_2_O_2_ in the cytosol, plastids, peroxisomes, and mitochondria, and the enzymes of the ascorbate–glutathione (GSH) pathway act in concert to remove H_2_O_2_ mainly in the cytosol and mitochondria, but probably also in other subcellular compartments (Dalton *et al.*, 1987, 1992; Iturbe-Ormaetxe *et al.*, 2001; Puppo *et al.*, 2005; Rubio *et al.*, 2007; Minguillón *et al.*, 2022). This latter pathway involves four enzymes: ascorbate peroxidase (APX), which reduces H_2_O_2_ to water using ascorbate as substrate; monodehydroascorbate reductase (MR) and dehydroascorbate reductase (DR), which reduce the oxidized forms of ascorbate; and glutathione reductase (GR), which regenerates GSH. Catalase and peroxiredoxins participate also in decomposition of H_2_O_2_, and glutathione peroxidases (Gpxs) reduce mainly lipid peroxides to the corresponding alcohols. Gpxs and peroxiredoxins use thioredoxins as electron donors and may also act as redox sensors in plant cells (Dietz *et al.*, 2006; Navrot *et al.*, 2006; Passaia and Margis-Pinheiro, 2015). As for the RNS, the mechanisms for the synthesis and scavenging of NO in plant cells have been extensively studied, although there are still several uncertainties, such as the elusive arginine-dependent NO synthase-like activity. This signaling molecule is synthesized through oxidative and reductive pathways and conveys its bioactivity mainly through *S*-nitrosylation of proteins; its concentration is kept under control by *S*-nitrosoglutathione reductase and phytoglobins (Glbs). For further information, see recent reviews by Lindermayr (2018), Larrainzar *et al.* (2020), and Gupta *et al.* (2022).

Recently, reactive sulfur species (RSS), of which hydrogen sulfide (H_2_S) is the most conspicuous member, have emerged as novel signaling molecules in animals and plants. RSS have important functions in development and in the stress response of plants (Aroca *et al.*, 2018; Hancock, 2019). In plant cells, H_2_S can be generated in the chloroplast by sulfite reductase during sulfate assimilation. The cytosol contains the highest cysteine (Cys) concentration in the cell and is a major source of H_2_S through the activities of l-Cys desulfhydrase, which releases H_2_S, pyruvate, and ammonia during l-Cys degradation, and d-Cys desulfhydrase, which is specific for d-Cys and occurs in some plant species. H_2_S is also generated in the mitochondria by β-cyanoalanine synthase during cyanide detoxification (Gotor *et al.*, 2019). At physiological pH, H_2_S exists mainly as the monoanion HS^−^ and hence we will refer here to H_2_S as the mixture of the two forms.

A major mechanism by which ROS, RNS, and RSS fulfil their signaling role is the post-translational modification (PTM) of proteins (Friso and van Wijk, 2015; Gotor *et al.*, 2019; Matamoros and Becana, 2021). H_2_S-induced persulfidation involves the conversion of the thiol group (R-SH) of reactive Cys residues into a persulfide (R-SSH). Persulfides show increased nucleophilicity compared with the thiol group and, as a consequence, the modified Cys may be more reactive and induce changes in protein function. Because the direct reaction of H_2_S and thiols is thermodynamically unfavorable and requires an oxidant (Filipovic *et al.*, 2018), potential mechanisms for persulfidation include the nucleophilic attack of H_2_S on oxidized protein thiols, such as sulfenic acids (R-SOH) and disulfide bridges, as well as on *S*-glutathionylated and *S*-nitrosylated Cys residues (Mishanina *et al.*, 2015).

Many legumes are able to establish symbiosis with soil bacteria collectively known as rhizobia. The onset of symbiosis entails a molecular dialog between the two partners that leads to the formation of N_2_-fixing root nodules (Udvardi and Poole, 2013). All stages of the symbiosis, from root infection to nodule maturation and senescence, require fine-tune signaling by ROS and RNS (Puppo *et al.*, 2005; Becana *et al.*, 2010; Bruand and Meilhoc, 2019; Matamoros and Becana 2020). More recently, it has been shown that H_2_S is produced in legume nodules (Fukudome *et al.*, 2020) and plays a beneficial role in symbiotic nitrogen fixation (SNF) (Zou *et al.*, 2020). However, nothing is known about the PTMs induced by H_2_S and other RSS. Here, we have examined the persulfidome of common bean (*Phaseolus vulgaris*) nodules at different developmental stages to gain insight into the functions of H_2_S in nodules of a major agricultural legume species. Our study comprised both the nodule host cells and the bacteroids. We conclude that H_2_S is an important player in the metabolic regulation of the two symbiotic partners and suggest that the function of H_2_S and persulfidation is to protect nodule proteins from oxidative modifications that lead to nodule senescence.

## Materials and methods

### Biological materials and plant treatments

Common bean (*Phaseolus vulgaris* cv. ‘Contender’) seeds were surface sterilized with 70% (v/v) ethanol and germinated in pots containing a perlite:vermiculite (1:1, v/v) mixture. After 1 week, seedlings were inoculated with *Rhizobium leguminosarum* bv. *phaseoli* strain 3622 and were grown on a nutrient solution containing 0.25 mM NH_4_NO_3_ in a controlled-environment chamber with a day/night regime of 23 °C/21 °C, 200 µmol photons m^−2^ s^−1^, and 16 h photoperiod (Loscos *et al.*, 2008). This small concentration of combined nitrogen does not inhibit nodulation and instead favors plant growth. Nodules were harvested from plants at three different developmental stages (days after germination): young (∼28 d, late vegetative stage), mature (∼40 d, late flowering–early fruiting stage), and senescent (∼53 d, fully developed pods). Nodules were kept at −80 °C until use.

*Lotus japonicus* seeds (ecotype MG-20) were gently scarified, surface sterilized, placed on 0.5% agar plates at 4 °C for 2–3 d, and germinated at 23 °C for 3 d in the dark. Seedlings were transferred to pots containing vermiculite, inoculated with *Mesorhizobium loti* strain MAFF303099, and watered twice a week with B&D nutrient solution supplemented with 0.25 mM NH_4_NO_3_ (Rubio *et al.* 2019). Plants were grown in a controlled-environment chamber in the same conditions as before. For measurement of nitrogenase activity, estimated as acetylene reduction activity (ARA), two independent experiments were performed in which *L. japonicus* plants were treated with the H_2_S donor sodium hydrogen sulfide (NaHS). In the first experiment, plants at 6 weeks post-inoculation (wpi) were treated with 0 (control) or 100 µM NaHS for 2 weeks and ARA was measured at 4, 6, and 8 wpi. In the second one, plants at 6 wpi were treated with 0 (control) or 100 µM NaHS for 4 weeks and ARA was measured at 6, 8, and 10 wpi.

### Expression and purification of recombinant proteins

Expression and purification of cowpea (*Vigna unguiculata*) iron-superoxide dismutase (VuFeSOD; GeneBank AAF28773.1) and *Lotus japonicus* glutathione peroxidase (LjGpx3; Lotus Base: LotjaGi4g1v0458000) were carried out as described by Moran *et al.* (2003) and Matamoros *et al.* (2015), respectively. Briefly, PCR fragments encoding the mature VuFeSOD and LjGpx3 proteins were cloned into pET-28a(+) (Novagen) and pET200/D-TOPO, respectively. His-tagged proteins were produced in *Escherichia coli* BL21 (DE3), purified on HiTrap chelating HP Ni-affinity columns (GE Healthcare Life Sciences), desalted, and concentrated by ultrafiltration. *L. japonicus* phytoglobin (LjGlb3-1; Lotus Base: Lj1g3v2035270.1) was cloned into pET11a (Novagen) with an N-terminal Strep-tag and was expressed in *E. coli* C41(DE3) cells (Lucigen) as described (Villar *et al.*, 2018). The Strep-tagged protein was purified on a StrepTactin Sepharose High Performance column (GE Healthcare), desalted, and concentrated by ultrafiltration.

### Protein persulfidation labeling by the tag-switch method

Biotinylation of endogenously persulfidated proteins was carried out as described previously (Zhang *et al.*, 2014; Aroca *et al.*, 2017). Young, mature, and senescent nodules (1 g) were ground in liquid nitrogen in a mortar and the proteins were isolated employing the TRIzol (Thermo Fisher Scientific) protocol as described by the manufacturer. One milligram of the protein extract was incubated with 50 mM 2-methylsulfonyl-benzothiazole (MSBT) in 50 mM Tris–HCl (pH 8.0) with 2.5% SDS at 37 °C for 60 min. Samples were precipitated with acetone to remove unreacted MSBT (1:5 v/v at –20 °C overnight) and were washed twice with cold 100% acetone and once with cold 70% acetone. The precipitated protein pellets were resuspended in 1 ml of 50 mM Tris–HCl (pH 8.0) buffer containing 2.5% SDS with 20 mM CN-biotin, and further incubated for 4 h at 37 °C. Reagents were then removed again by acetone precipitation, and after washes, the pellets were resuspended in 200 µl of 50 mM Tris–HCl (pH 8.0) buffer with 150 mM NaCl. To purify the labeled proteins, the solution was incubated with 150 µl of magnetic streptavidin-coated particles (Sera-Mag, Cytiva, UK) for 1 h at room temperature in a rotatory mixer. Then, the beads were intensively washed with a buffer containing 50 mM Tris–HCl (pH 8.0), 600 mM NaCl, 1 mM EDTA, and 0.5% Triton X-100, at room temperature within a magnetic rack. Bound proteins were eluted with a solution of 2% SDS, 30 mM biotin, 50 mM phosphate, 100 mM NaCl, 6 M urea, and 2 M thiourea for 15 min at room temperature, followed by an incubation of 15 min at 96 °C. As controls, sample aliquots were incubated with 100 mM dithiothreitol (DTT) for 1 h at 37 °C prior to tag-switch labeling. The proteins were then acetone precipitated for nano-liquid chromatography–tandem mass spectrometry (nLC-MS/MS) analysis.

### Label-free protein quantification by SWATH-MS acquisition and analysis

Protein concentration from eluted streptavidin beads was determined using a Pierce 660 nm protein assay (Thermo Fisher Scientific). An aliquot of 50 μg protein was resuspended in 50 mM ammonium bicarbonate with 0.2% Rapigest (Waters) and digested with sequencing-grade modified trypsin (Sigma-Aldrich) at 37 °C overnight on a shaker. Three biological replicates for each nodule developmental stage were analysed. A data-dependent acquisition (DDA) approach using nLC-MS/MS was first performed to generate a SWATH (sequential window acquisition of all theoretical fragment ion spectra mass spectrometry)–MS spectral library as described (García *et al.*, 2019).

The peptide and protein identifications were performed using Protein Pilot software (version 5.0.1, Sciex) with the Paragon algorithm. The search was conducted against *Phaseolus vulgaris* and *Rhizobium leguminosarum* Uniprot proteome databases, specifying acetyl (protein N-terminus), CN-Biotin-Na-Sulfide (Cys), CN-Biotin-Sulfide (Cys), Methylthio (Cys), MSBT (Cys), Oxidation (methionine), and Sulfide (Cys) as variable modifications. The false discovery rate (FDR) was set to 0.01 for both peptides and proteins. The MS/MS spectra of the identified peptides were then used to generate the spectral library for SWATH peak extraction using the add-in for PeakView Software (version 2.1, Sciex) MS/MSALL with SWATH Acquisition MicroApp (version 2.0, Sciex). Peptides with a confidence score >99% (as obtained from the Protein Pilot database search) were included in the spectral library.

For relative quantification using SWATH analysis, the same samples used to generate the spectral library were analysed using a data-independent acquisition (DIA) method. Each sample (2 μl) was analysed using the LC-MS equipment and LC gradient described above to build the spectral library but using instead the SWATH-MS acquisition method. The method consisted of repeating an acquisition cycle of time of flight (TOF) MS/MS scans (230–1500 *m/z*, 60 ms acquisition time) of 60 overlapping sequential precursor isolation windows of variable width (1 *m/z* overlap) covering the 400–1250 *m/z* mass range with a previous TOF MS scan (400 to 1250 *m/z*, 50 ms acquisition time) for each cycle. The total cycle time was 3.7 s.

The targeted data extraction of the fragment ion chromatogram traces from the SWATH runs was performed by PeakView (version 2.1) with the MS/MSALL with SWATH Acquisition MicroApp (version 2.0). This application processed the data using the spectral library created from the shotgun data. Up to 10 peptides per protein and seven fragments per peptide were selected, based on signal intensity. Any shared and modified peptides were excluded from the processing. Windows of 12 min and 20 ppm width were used to extract the ion chromatograms. SWATH quantification was attempted for all proteins in the ion library that were identified by Protein Pilot with an FDR <1%. The extracted ion chromatograms were then generated for each selected fragment ion. The peak areas for the peptides were obtained by summing the peak areas from the corresponding fragment ions. PeakView computed an FDR and a score for each assigned peptide according to the chromatographic and spectra components. Only peptides with an FDR <5% were used for protein quantification. Protein quantification was calculated by adding the peak areas of the corresponding peptides. To test for differential protein abundance between the two groups, MarkerView (version 1.2.1, Sciex) was used for signal normalization.

### Identification of persulfidated Cys residues of recombinant proteins using MS

Recombinant proteins (~50 µg) were treated with 200 µM NaHS for 30 min at room temperature and precipitated with trichloroacetic acid–acetone (1:8 v/v per volume of sample) after treatment. Recombinant proteins without NaHS treatment were also analysed. Precipitated proteins were resuspended in a solution of RapidGest 0.2% (w/v) in 50 mM ammonium bicarbonate and 10 mM chloroacetamide. Samples were incubated for 30 min in the dark at room temperature. The proteins were finally digested with trypsin at 1:40 trypsin: protein (w/w) overnight at 37 °C. Digestion was stopped by the addition of formic acid and acetonitrile to 2% final concentration. Digested peptides were subjected to one-dimensional nLC-MS/MS with electrospray ionization (nanoLC Ultra 1D plus; Eksigent Technologies) coupled to a TripleTOF 5600 mass spectrometer (Sciex) with a duo spray ionization source. Data acquisition was performed using a TripleTOF 5600 System (Sciex). MS and MS/MS data obtained for individual samples were processed using Analyst TF 1.5.1 software (Sciex). The identification of the proteins was carried out with an ‘in-house Mascot 2.6 search engine’ including as variable modification: Carbamidomethyl (C), S_Cam (C), Oxidation (M), and Sulfide (S).

### Assay of enzyme activities

All antioxidant enzymes were extracted at 0 °C and assayed at 25 °C within a linear range. The enzymes were extracted from 100 mg of nodules using 0.4 ml of the following optimized media. APX: 50 mM KP_i_ buffer (pH 7.0), 0.5% (w/v) polyvinylpyrrolidone-10; MR, DR, and GR: 50 mM KP_i_ buffer (pH 7.8), 1% polyvinylpyrrolidone-10, 0.2 mM EDTA, and 10 mM β-mercaptoethanol; l-galactono-1,4-lactone dehydrogenase (GalLDH): 50 mM Tris–HCl (pH 8.0), and 0.15% (v/v) Triton X-100. All extracts were centrifuged at 20 000 *g* for 10 min at 4 °C, and the enzyme activities were assayed in the supernatants. To investigate the effect of H_2_S on enzyme activity, supernatants were treated with different concentrations of NaHS (0–500 μM) at 26 °C for 1 h. APX and DR activities were determined by following ascorbate oxidation at 290 nm (Asada, 1984) and ascorbate formation at 265 nm (Nakano and Asada, 1981), respectively. MR and GR activities were assayed by following the oxidation of NADH and NADPH (Loscos *et al.*, 2008) at 340 nm, respectively. GalLDH activity was determined by following the reduction of cytochrome *c* at 550 nm (Bartoli *et al.*, 2000).

Nitrogenase activity was assessed as ARA. To this end, immediately after harvest, nodules were introduced into glass bottles sealed with rubber stoppers. Acetylene (2 ml) was injected into each bottle after the same volume of air was pumped out. All bottles were subsequently incubated for 2 h at 28 °C. Samples of 100 µl of gas from each bottle were used to measure ethylene production using a GC-4000A gas chromatograph (East & West Analytical Instruments, Beijing, China). Five biological replicates were performed and each replicate contained nodules from five plants.

### Analysis of protein nitration, oligomerization, and redox state

The oligomerization and redox state of LjGpx3 and LjGlb3-1 was analysed by incubating the recombinant proteins (20 μM) with either 500 μM DTT or 200–500 μM H_2_O_2_ in the presence or absence of 500 μM NaHS for 1 h at 30 °C in 50 mM KP_i_ buffer (pH 7.4). The proteins were then separated on 15% SDS-PAGE and analysed by Coomassie Blue staining and immunoblots using specific antibodies against LjGpx3 and LjGlb3-1. Nitration of recombinant VuFeSOD, LjGpx3, and LjGlb3-1 was done by mixing the proteins (20 μM) with 500 μM 3-morpholino-sydnonimine (SIN-1) for 2 h at 30 °C. The proteins were then separated on 15% SDS-PAGE and analysed by immunoblotting using specific polyclonal antibodies against VuFeSOD, LjGpx3, and LjGlb3-1, and a monoclonal anti-NO_2_–Tyr antibody (Cayman Chemical Co.). Immunoreactive proteins were detected by chemiluminescence using the SuperSignal West Pico kit (Thermo Fisher Scientific).

## Results

### Identification of persulfidated proteins in legume nodules

In this study we have used the tag-switch method, which specifically labels persulfidated proteins with CN-biotin (Aroca *et al.*, 2017), to uncover the *in vivo* persulfidation profile of common bean nodule proteins. Nodules were harvested from plants at three different stages of development. These nodules were termed ‘young’, ‘mature’, and ‘senescent’ and correspond, respectively, to 3, 5, and 7 wpi. In total, we identified 967 proteins of the host cells (Supplementary Tables S1–S7) and 409 proteins of bacteroids (Supplementary Tables S8, S9) (FDR<0.01) with altered persulfidation levels at the different developmental stages. A 1.5-fold change in protein abundance was used as the threshold value, with a significance level of *P*<0.05. The peptides used for protein identification are provided in Supplementary Datasets S1–S3.

**Fig. 1.**
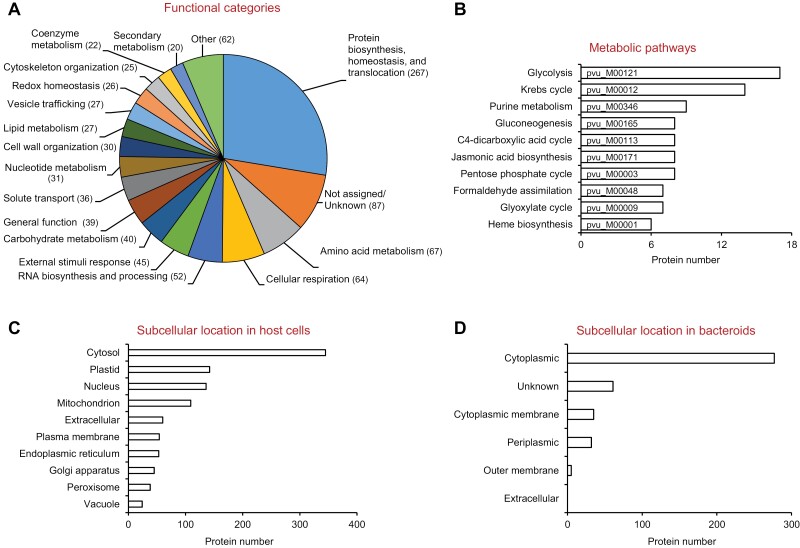
Functional classification and predicted subcellular location of plant and bacteroid persulfidated proteins. (A) Number of persulfidated plant proteins in different functional categories. Common bean nodule proteins were BLASTed against the Arabidopsis and *Lotus japonicus* proteomes. The closest homologs were classified using Mercator4. (B) Distribution of unigenes coding for persulfidated proteins to KEGG modules. The 10 KEGG module identifiers with the highest numbers of unigenes are shown. (C) Predicted subcellular location of persulfidated plant proteins. The subcellular location of Arabidopsis closest homologs was determined according to the SUBA database. (D) Predicted subcellular location of persulfidated bacteroid proteins according to PSORTb version 3.0.

To gain insight into the cellular processes that may be regulated by persulfidation in nodules, the datasets containing the plant proteins were mined with the software tools Mercator4 (Schwacke *et al.*, 2019), Kyoto Encyclopedia of Genes and Genomes (KEGG; Kanehisa *et al.*, 2022), and AgriGO (Tian *et al.*, 2017). It should be noted that, in these analyses, low abundant proteins and their associated pathways might be overlooked due to bias for the detection of more abundant peptide species by MS. Persulfidated proteins were classified into 29 functional categories. A total of 87 proteins could not be assigned to any category because of their unknown function. The categories with a higher number of persulfidated proteins were those related to the biosynthesis, homeostasis, modification, and translocation of proteins (267), followed by amino acid metabolism (67), cellular respiration (64), and RNA biosynthesis and processing (52) (Fig. 1A). To identify functional units in metabolic pathways, the proteins were mapped using the KEGG tools. The analysis suggests that the pathways of glycolysis, tricarboxylic acid cycle, purine metabolism, and C_4_-dicarboxylic acid cycle are potentially regulated by H_2_S (Fig. 1B). Dicarboxylic acid metabolism is very important for SNF because malate is the major form in which photosynthesis-derived carbon is transported into bacteroids, where it is oxidized to produce ATP through respiration (Udvardi and Poole, 2013). To determine whether genes coding for persulfidated proteins preferentially clustered into specific gene ontology (GO) categories, a singular enrichment analysis was carried out with the AgriGO tool. A total of 55 GO terms were significantly over-represented (FDR<0.001) in the biological process domain (Table 1; Supplementary Fig. S1).

**Table 1.** Singular enrichment analysis of common bean genes coding for persulfidated proteins in GO categories of biological processes

GO terms	Description	% Persufidated*^a^*	%/Ref*^b^*	FDR
GO:0019752	Carboxylic acid metabolism	8.6	2.3	2.92 × 10^−16^
GO:0006520	Amino acid metabolism	7.0	1.5	2.92 × 10^−16^
GO:0006412	Translation	8.9	3.1	9.10 × 10^−12^
GO:0055086	Nucleotide metabolism	4.7	1.2	1.74 × 10^−9^
GO:0006096	Glycolysis	1.9	0.3	8.43 × 10^−6^

a Percentage of genes coding for persulfidated proteins in the biological process.

b Percentage of genes in each GO category in the *Phaseolus vulgaris* database.

Biological membranes are expected to slow down H_2_S transport between cellular compartments (Filipovic *et al.*, 2018). Consequently, the site of H_2_S synthesis is relevant for its signaling activity. We analysed the subcellular distribution of the identified persulfidated proteins. First, common bean proteins were BLASTed against the Arabidopsis proteome and the subcellular location of the closest homologs was determined according to the SUBA database (Hooper *et al.*, 2017). Most of the persulfidated proteins identified in this study are located in the cytosol (34%), followed by the nucleus (14%), plastid (14%), and mitochondrion (11%) (Fig. 1C). This is consistent with the observations that the cytosol contains by far the highest Cys concentration in plant cells and that several enzymes that catabolize Cys and release sulfide are located in that compartment (Gotor *et al.*, 2019). In common bean nodules, a gene coding for l-Cys desulfhydrase is expressed at relatively high levels in young and mature nodules (O’Rourke *et al.*, 2014), suggesting that this activity generates H_2_S in the cytosol of nodule cells and thus contributes to protein persulfidation.

Bacterial proteins in nodules were investigated using Clusters of Orthologous Groups (COG; Galperin *et al.*, 2015) to determine which cellular processes are associated to a higher proportion of persulfidated proteins. Four categories, including carbohydrate, lipid, amino acid, and nucleotide transport and metabolism, were found to be enriched in modified proteins (Fisher’s exact test, *P*< 0.01; Table 2), suggesting a role for persulfidation in core bacteroid metabolism. Remarkably, nitrogenase components were detected as persulfidated (Supplementary Table S8), indicating that H_2_S may have also a direct effect on SNF. According to PSORTb (Yu *et al.*, 2010), a significant high proportion of persulfidated bacteroidal proteins (68%) locates to the cytosol (Fisher’s exact test, *P*<0.01; Fig. 1D), where H_2_S can be produced by sulfite reductase and cystathionine γ-lyase (Becana *et al.*, 2018; Zou *et al.*, 2020).

**Table 2.** Enrichment analysis of *Rhizobium leguminosarum* genes coding for persulfidated proteins into functional categories (COG)

COG	% Persufidated*^a^*	%/Ref*^b^*	*P* value
Carbohydrate transport and metabolism	11.7	5.0	1.00 × 10^−5^
Lipid transport and metabolism	5.1	2.5	3.80 × 10^−3^
Amino acid transport and metabolism	14.1	5.6	1.0 × 10^−5^
Nucleotide transport and metabolism	5.4	2.4	1.1 × 10^−3^
Mobilome: prophages, transposons	0.0	2.4	1.00 × 10^−4^
Cell motility	0.0	1.8	1.70 × 10^−3^

a Percentage of genes coding for persulfidated proteins in the biological process.

b Percentage of genes in each COG category in *Rhizobium leguminosarum* database.

### Persulfidation decreases with nodule senescence in the host cells but not in the bacteroids

Quantitative changes in the persulfidation levels of proteins were determined at three developmental stages of nodules to better understand the role of H_2_S in nodule development and senescence. Nodule aging was generally accompanied by a decrease in persulfidation levels. The number of modified plant proteins found exclusively in young nodules was 181 (Supplementary Table S1), whereas 115 proteins were detected only in mature nodules (Supplementary Table S3) and 31 proteins in senescent nodules (Supplementary Table S6). The transition from the mature to the senescent stage involved the strongest decline in the persulfidation status, with a total of 502 plant proteins showing a quantitative reduction in persulfidation as the nodules aged (Supplementary Table S5). In contrast, only 82 proteins had higher levels of persulfidation in senescent nodules relative to mature nodules (Supplementary Table S7).

**Fig. 2.**
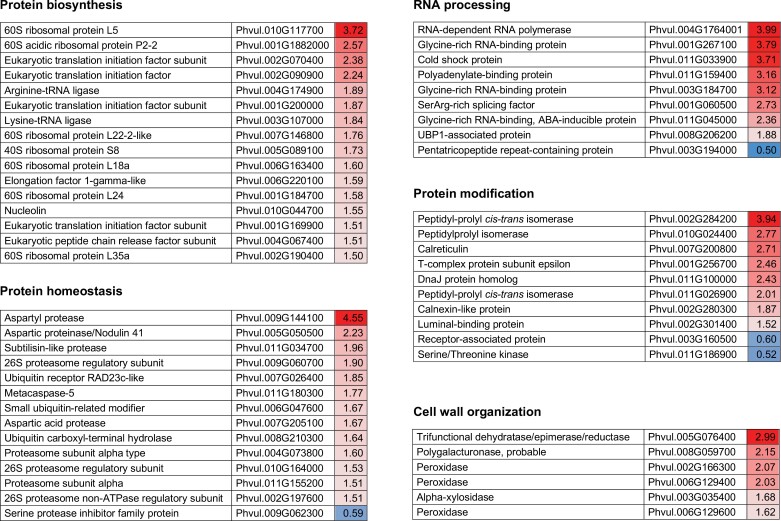
Quantitative changes in persulfidation of plant proteins during nodule development. For each protein, significant higher levels of persulfidation in young nodules compared with mature nodules are shown in different shades of red. Significant lower levels of persulfidation are shown in different shades of blue. Statistically significant differences (*P*<0.05) are based on analysis using the MarkerView software (Sciex).

**Fig. 3.**
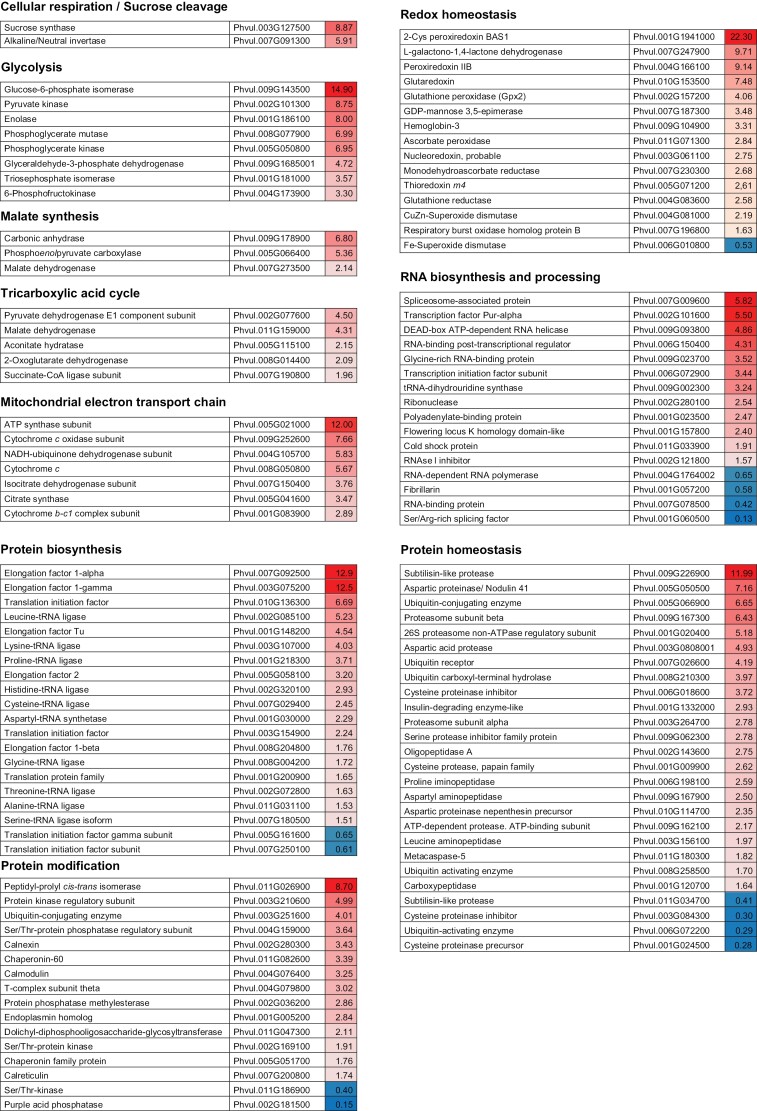
Quantitative changes in persulfidation of plant proteins during nodule senescence. For each protein, significant higher levels of persulfidation in mature nodules compared with senescent nodules are shown in different shades of red. Significant lower levels of persulfidation are shown in different shades of blue. Statistically significant differences (*P*<0.05) based on the analysis using the MarkerView software (Sciex).

During the transition from young to mature nodules, the functional categories that showed major reductions in protein persulfidation were RNA processing, protein synthesis, protein homeostasis and modification, and cell wall organization (Fig. 2; Supplementary Table S2). Similarly, the transition of mature to senescent nodules also correlated with a sharp decline in the persulfidation levels of proteins involved in protein and RNA metabolism, as well as of proteins that participate in redox homeostasis, cell respiration, and the synthesis of malate for delivery into the bacteroids (Fig. 3; Supplementary Table S5). These results indicate that a decrease in persulfidation in senescent nodules may be linked to a lower capacity to maintain redox homeostasis and metabolic activity, as observed in animal systems (Zivanovic *et al.*, 2019). Interestingly, several proteins involved in H_2_O_2_ and NO homeostasis and in ascorbate biosynthesis were identified as targets of persulfidation, mainly in mature nodules. In contrast, two FeSODs and one cytosolic Gpx showed highest levels of persulfidation in senescent nodules (Fig. 3; Supplementary Tables S6, S7). These findings reflect a crosstalk between H_2_S, ROS, and NO signals that could be important in the aging process. Notably, one of the FeSOD isoforms is a homolog of an unusual cytosolic FeSOD of cowpea nodules, which has been related to nodule senescence (Moran *et al.*, 2003). Our results suggest a possible effect of persulfidation on the function of this FeSOD isoform (see under heading ‘Persulfidation interferes with protein nitration’).

**Fig. 4.**
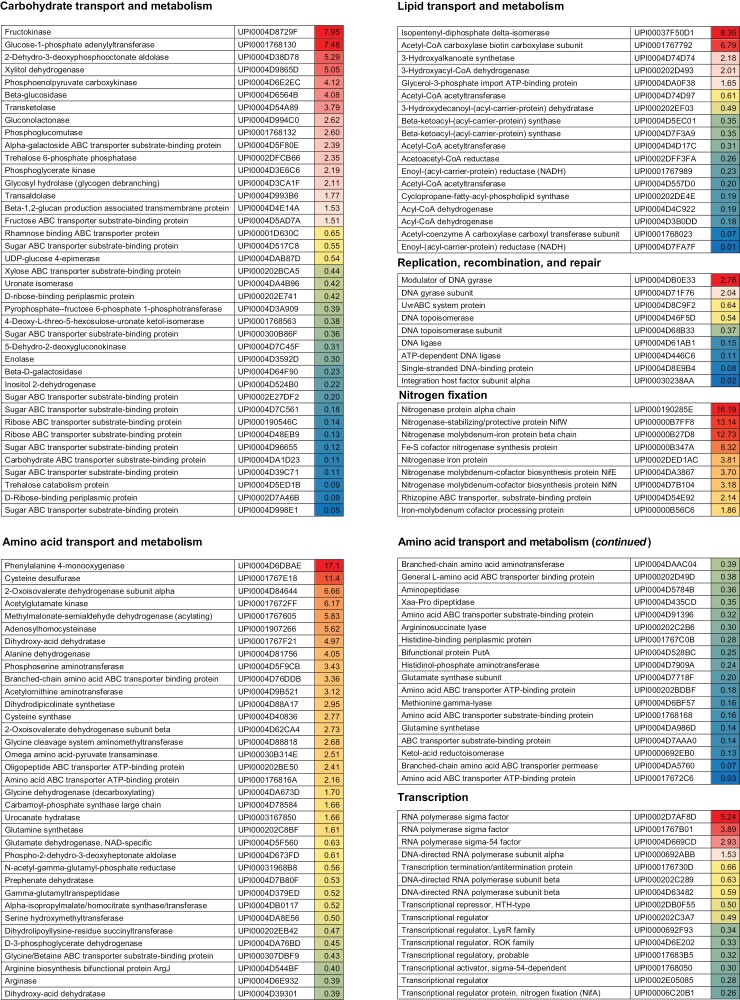

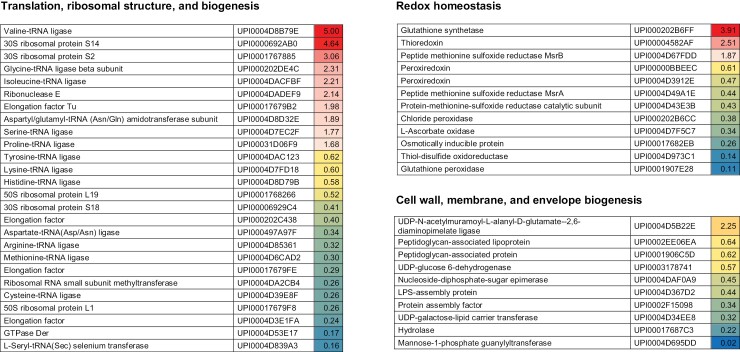
Quantitative changes in persulfidation of bacteroid proteins during nodule senescence. For each protein, significant higher levels of persulfidation in young and mature nodules compared with senescent nodules are shown in different shades of red. Significant lower levels of persulfidation are shown in different shades of blue. Statistically significant differences (*P*<0.05) based on the analysis using the MarkerView software (Sciex).

The persulfidation levels of bacteroids were higher in senescent nodules than in young or mature nodules. A total of 158 proteins of bacteroids were found to be more persulfidated in young and mature nodules than in senescent nodules, whereas 251 proteins of bacteroids showed maximum levels of persulfidation in senescent nodules (Supplementary Tables S8, S9). Many proteins belonging to functional categories related to carbohydrate, lipid, and amino acid transport and metabolism showed increased protein persulfidation in the bacteroids of senescent nodules. This was also the case for proteins involved in replication, transcription, translation, and redox homeostasis, as well as in the biogenesis of cell wall, membrane, and envelope of bacteroids. These findings indicate that bacteroids from senescent nodules are metabolically active (Fig. 4; Supplementary Tables S8, S9). However, all the detected nitrogenase components were more persulfidated in young and mature nodules than they were in senescent nodules (Fig. 4).

### Persulfidation may regulate enzyme activities involved in ROS homeostasis in nodules

A few studies support the existence of a crosstalk between H_2_O_2_ and H_2_S in the regulation of natural senescence and in the stress response of plants and animals (Aroca *et al.*, 2018; Zivanovic *et al.*, 2019). One way in which H_2_S may interact with other redox signals is the persulfidation of proteins involved in regulation of intracellular H_2_O_2_ levels and thereby of their bioactivity. To test this hypothesis, we selected enzymes involved in redox homeostasis that are persulfidated *in vivo*, and investigated the possible effect of NaHS treatment on their bio-logical function. NaHS is commonly used as an H_2_S donor in plants and animals and has been shown to induce persulfidation in a dose-dependent manner (S. Chen *et al.*, 2020). However, it should be noted that the concentration of H_2_S is difficult to estimate because in solution NaHS is readily hydrolysed to H_2_S and HS^−^ and because part of H_2_S is released to the gas phase. To circumvent these problems, experiments were carried out at different concentrations of NaHS and slightly basic pH and in the presence of metal chelators to stabilize H_2_S and avoid its oxidation (Filipovic *et al.*, 2018). To induce persulfidation, incubations were carried out for 1 h at 26 °C. Then, excess NaHS was removed using desalting columns.

**Fig. 5.**
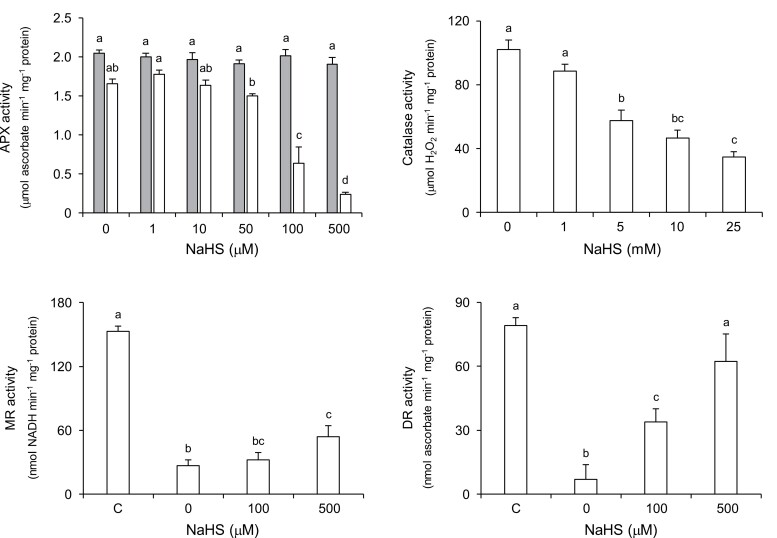
Specific activities of the enzymes of the ascorbate–glutathione pathway and catalase in common bean nodules. Persulfidation of proteins was induced by incubation with different concentrations of hydrogen sulfide (supplied as NaHS) for 1 h at 26 °C. APX activity was assayed in samples in which persulfidation was induced in the presence (gray bars) or absence (white bars) of 5 mM ascorbate. For MR and DR activities, controls (C) show the enzymatic activities obtained in assays carried out immediately after tissue homogenization in the presence of 10 mM β-mercaptoethanol. Values are means ±SE of four to eight replicates. Means denoted by the same letter are not significantly different at *P*<0.05 based on Duncan’s multiple range test. APX, ascorbate peroxidase; DR, dehydroascorbate reductase; MR, monodehydroascorbate reductase.

We have detected persulfidated versions of catalase and three enzymes of the ascorbate–GSH cycle. In all cases, the persulfidation level of the proteins decreased in senescent nodules (Fig. 3). Because this finding may be relevant for the regulation of nodule senescence, we assessed the effect of this PTM on protein activity. Total APX activity was determined in nodule extracts treated with different concentrations of NaHS (0–500 μM). Our results show that low concentrations (1–50 μM) do not modify enzyme activity (Fig. 5A), although a positive effect of NaHS on APX activity, which was attributed to persulfidation of the protein at Cys-32, was reported in Arabidopsis leaf extracts and in recombinant Arabidopsis cytosolic APX1 (Aroca *et al.*, 2015; Kaur *et al.*, 2021). In nodules, cytosolic APX, which is by far the major isoform, is stable in the absence of ascorbate, contrary to the chloroplastic isoforms (Dalton *et al.*, 1987), indicating slight structural differences between nodule and leaf enzymes. Remarkably, the APX activity of nodules was inhibited by high concentrations (>50 μM) of NaHS and this inhibition was completely abolished in the presence of ascorbate (Fig. 5A). There are at least two explanations: (i) excess H_2_S is oxidized by APX to the sulfiyl radical (HS•) that inactivates the enzyme (Chen and Asada, 1992); in this case, ascorbate would scavenge HS• (Filipovic *et al.*, 2018) protecting APX activity; and (ii) binding of H_2_S to the heme of APX, as occurs with myoglobin (Jensen and Fago, 2018), which would probably inhibit enzyme activity; in this case, ascorbate would interfere with H_2_S binding at the catalytic site. Unfortunately, our data cannot differentiate between the two mechanisms, which may operate simultaneously.

Unlike APX, catalase activity in nodule extracts was unaffected by 0–500 μM of NaHS and much greater concentrations (up to 5 mM) were necessary to cause significant inhibition (Fig. 5B). However, the implication of Cys persulfidation in the inhibition of catalase activity is difficult to establish unequivocally because it might be caused by the interaction of H_2_S with the heme (Filipovic *et al.*, 2018). In nodules, a regulatory effect of H_2_S on catalase activity is unlikely because high concentrations of H_2_S are required for inhibition.

The enzymes MR and DR contain labile thiols that are essential for enzyme activity and are rapidly inactivated in the absence of thiol protectants (Dalton *et al.*, 1992). Indeed, MR activity was only 17% of that obtained in the presence of 10 mM β-mercaptoethanol after 1 h if a reductant was not included in the extraction medium. However, when 500 μM NaHS was present during incubation, 35% of the activity was retained (Fig. 5C). These results indicate that persulfidation of MR might have a protective function under conditions in which thiols are oxidized leading to the inactivation of the enzyme. In the case of DR, formation of sulfenic acid occurs at the catalytic Cys during the reduction of dehydroascorbate and under oxidative conditions. The irreversible overoxidation of the sulfenic group to sulfinic or sulfonic acid can be prevented by *S*-glutathionylation of the enzyme (Bodra *et al.*, 2017). Our results indicate that H_2_S might fulfil a similar protective function. Thus, ~90% of DR activity was lost after 1 h incubation, probably due to enzyme oxidation. However, inactivation was almost completely prevented by NaHS (Fig. 5D). In contrast, we could not observe significant changes in GR activity comparing extracts incubated with or without NaHS (data not shown).

Two proteins involved in ascorbate biosynthesis, GDP-mannose 3,5-epimerase and l-galactono-1,4-lactone dehydrogenase (GalLDH), were found to be persulfidated *in vivo* (Supplementary Table S5). In both enzymes, the persulfidation levels were lower in senescent nodules, consistent with the decreases in GalLDH activity and ascorbate content previously reported (Loscos *et al.*, 2008). GalLDH contains redox sensitive thiols and is inactivated by selective oxidation of one of its Cys residues (Leferink *et al.*, 2009). Hence, H_2_S and protein persulfidation might participate in the regulation of ascorbate biosynthesis in nodules. To test this possibility, we determined the ascorbate content of nodules of 4-week-old plants grown for 2 weeks in the presence or absence of 100–500 μM NaHS. We also measured GalLDH activity in mitochondria-enriched fractions obtained from the same nodules and in mitochondrial fractions treated with NaHS. However, we could not observe a significant effect of NaHS on the ascorbate content or GalLDH activity in our experimental conditions (data not shown).

### A possible role of persulfidation in the regulation of the redox state and dimerization of proteins

In plants, transient perturbations of redox homeostasis provoke reversible and irreversible PTMs of proteins that may trigger structural alterations and signaling events, leading in some cases to oxidative damage. Because of its high nucleophilic capacity, Cys residues are major targets of redox-based PTMs (Waszczak *et al.*, 2018). To get insight into the possible ways by which H_2_S and protein persulfidation influence ROS- and NO-mediated signaling, we investigated the effect of this PTM on the redox and oligomerization states of two proteins, Gpx3 and Glb3-1, that we found to be persulfidated *in vivo* (Supplementary Tables S5, S6) and may participate in the modulation of ROS and RNS levels, respectively (Gupta *et al*., 2011; Matamoros *et al.*, 2015). In these experiments we used the orthologous proteins of the model legume *L. japonicus*, LjGpx3 and LjGlb3-1, for three reasons: in our laboratory we have set up optimized protocols for the production of both proteins at very high yield and purity, as required for *in vitro* experiments; the proteins of *L. japonicus* and common bean share high amino acid identity (86% and 77%, respectively); and all the Cys residues susceptible of persulfidation are conserved in the proteins of the two species (Supplementary Fig. S2). We treated recombinant LjGpx3 and LjGlb3-1 with NaHS and analysed their persulfidation state by MS to identify the modified Cys residues. We found that Cys-159 of LjGlb3-1 and Cys-81 of LjGpx3 are persulfidated (Supplementary Figs S3, S4). To minimize a direct interaction between H_2_S and H_2_O_2_, the proteins were treated with NaHS for 1 h, diluted 1:1 in reaction buffer, and incubated with H_2_O_2_. The experiments were carried out at 26 °C and pH 7.4 to minimize H_2_S decomposition (Mishanina *et al.*, 2015). Optimal assay conditions were established at 20 μM recombinant protein, 500 μM NaHS, and 200–500 μM H_2_O_2_.

**Fig. 6.**
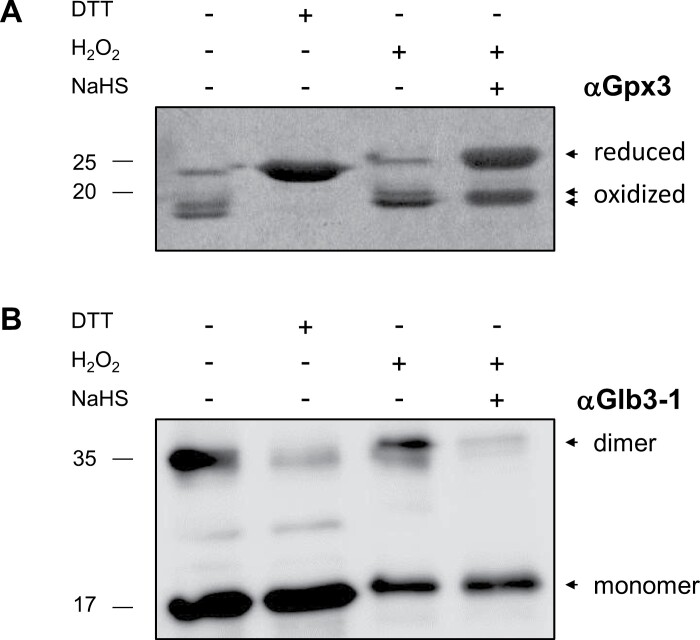
Effect of H_2_S (supplied as NaHS) on LjGpx3 and LjGlb3-1 redox state. Recombinant proteins (10 μM) were treated with reducing (500 μM DTT) or oxidizing (200–500 μM H_2_O_2_) agents in the presence or absence of NaHS (500 μM), and subjected to SDS-PAGE and immunoblot analyses using specific antibodies. The figure shows representative immunoblots from three independent experiments. Glb3, class 3 hemoglobin; Gpx, glutathione peroxidase.

In our purification conditions, LjGpx3 was mostly oxidized. Indeed, the migration pattern of the untreated and oxidized proteins was identical, showing the presence of two close bands corresponding to the oxidized protein (Fig. 6A). In contrast, under reducing conditions (500 μM DTT), the recombinant protein migrated at its theoretical molecular mass and no additional bands were observed. Interestingly, the preincubation of the protein with 500 μM NaHS, or the co-incubation with NaHS and H_2_O_2_, partially avoided enzyme oxidation. The most plausible explanation is that H_2_O_2_ oxidizes Cys residues of LjGpx3 leading to formation of sulfenic acids and/or disulfide bridges that react with H_2_S to produce persulfides.

Several Glbs have been shown to be involved in the regulation of intracellular NO (Gupta *et al.*, 2011; Larrainzar *et al.*, 2020). As was the case of LjGpx3, we treated recombinant LjGlb3-1 with NaHS and then with H_2_O_2_ to study its redox behavior. Under non-reducing conditions, LjGlb3-1 was found as monomer and dimer (Fig. 6B). Treatment with DTT provoked the almost total disappearance of the dimer, indicating that it was produced by disulfide bridges as observed for LjGlb3-2 (Rubio *et al.*, 2019). In contrast, incubation with H_2_O_2_ caused partial dimerization of the protein, whereas pretreatment with NaHS retained the protein in monomeric form even in the presence of H_2_O_2_. To assess if LjGlb3-1 exists predominantly as dimer or monomer *in vivo*, we analysed proteins of *L. japonicus* nodules in non-reducing SDS-PAGE gels, and bands encompassing proteins of 18–20 kDa or 36–40 kDa were excised from the gels for identification by nLC-MS/MS. In all cases, LjGlb3-1 was found in the 18–20 kDa region of the gel, suggesting that the monomer is predominant in nodules under physiological conditions.

### Persulfidation interferes with protein nitration

**Fig. 7.**
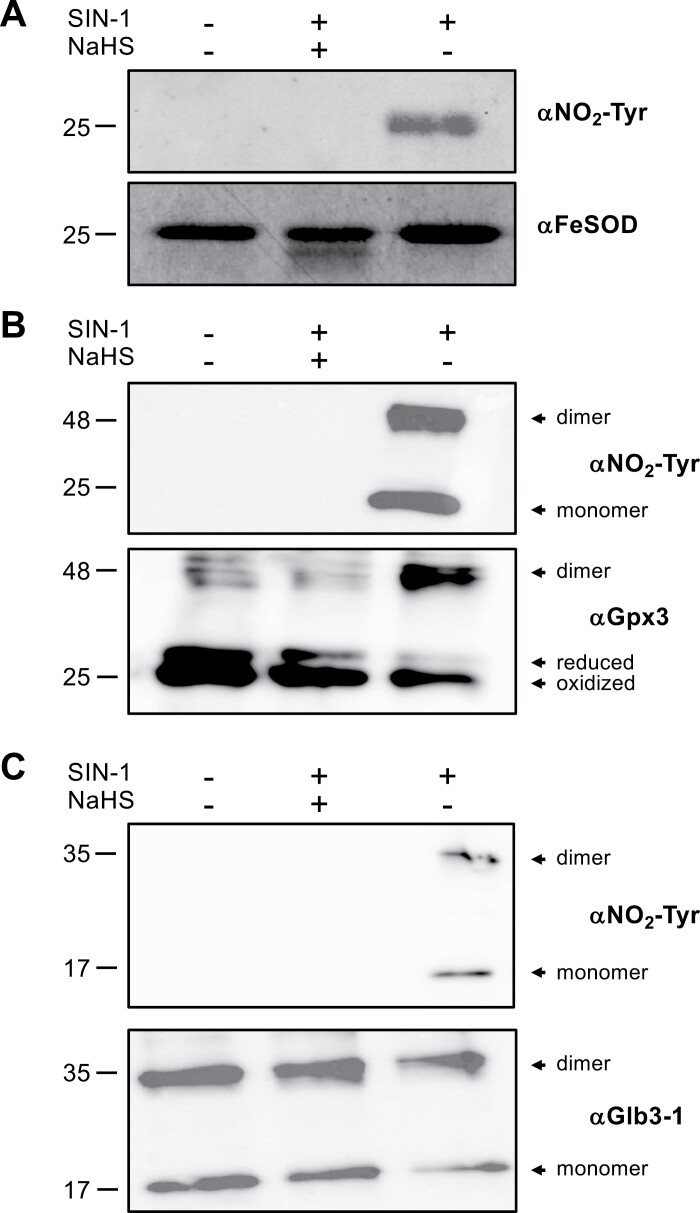
Effect of H_2_S (supplied as NaHS) on protein nitration. Nitration of VuFeSOD (A), LjGpx3 (B), and LjGlb3-1 (C) was induced by incubation of recombinant proteins (5–10 μM) with 500 μM SIN-1 for 2 h at 30 °C in 50 mM KPi (pH 7.4) in the presence or absence of 500 μM NaHS. The proteins were loaded on SDS gels, blotted, and probed with an anti-NO_2_–Tyr antibody and specific antibodies for each protein. Glb3, class 3 hemoglobin; Gpx, glutathione peroxidase; SOD, superoxide dismutase.

Tyrosine (Tyr) nitration is a RNS-mediated PTM that is linked to nitro-oxidative damage in plants and animals (Corpas *et al.*, 2013; Bartesaghi and Radi, 2018). In human recombinant MnSOD, Tyr nitration by ONOO^−^ was reduced in the persulfidated enzyme, suggesting that this PTM might serve as a protective mechanism against nitro-oxidative stress (Zivanovic *et al.*, 2019). To assess whether a similar mechanism is operative in plants, we used recombinant VuFeSOD, which is nitrated *in vitro* (Urarte *et al.*, 2014). This protein is homologous (94% identity; Supplementary Fig. S2) to common bean FeSOD (Phvul.007G135400), which was found to be persulfidated only in senescent nodules (Supplementary Table S6). We used SIN-1, an ONOO^−^ generator, as the nitrating agent. As expected, when VuFeSOD was incubated with 500 μM SIN-1 for 2 h at 30 °C, a single band of nitrated protein was detected on immunoblots using a monoclonal anti-NO_2_–Tyr antibody. However, preincubation with NaHS completely prevented nitration of VuFeSOD, indicating a protective function of H_2_S and persulfidation (Fig. 7A).

Common bean and *L. japonicus* Gpx3 and Glb3-1 contain conserved sites that are prone to nitration according to Tyr nitration predicting software iNitro-Tyr (Supplementary Fig. S5) (Xu *et al.*, 2014). In particular, LjGpx3 contains three putative nitration sites (Tyr-10, Tyr-27, and Tyr-48), whereas LjGlb3-1 has only one putative nitration site (Tyr-72). These predictions prompted us to assess the effect of NaHS treatment on the nitration profiles of both proteins. Treatment of LjGpx3 and LjGlb3-1 with SIN-1 induced protein nitration and oligomerization (Fig. 7B, C). Furthermore, in LjGlb3-1 we could detect nitrated monomers, dimers, and in some experiments, also high molecular mass (>100 kDa) aggregates. This aggregation triggered by RNS may share a similar mechanism to that found for leghemoglobins of soybean (*Glycine max*) and common bean, which was reported by our group (Sainz *et al.*, 2015). However, pretreatment with NaHS completely abolished protein nitration of LjGpx3 and LjGlb3-1, suggesting that H_2_S and persulfidation protect the proteins from this nitro-oxidative modification.

### H_2_S delays nodule senescence

**Fig. 8.**
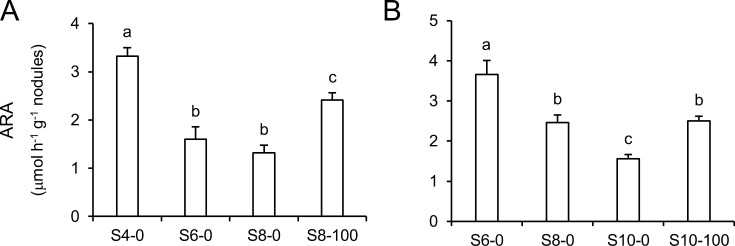
Effect of H_2_S (supplied as NaHS) on nitrogenase activity (ARA) of *Lotus japonicus* nodules. (A) Nitrogenase activity was measured in nodules at 4 wpi (young; S4-0), 6 wpi (mature; S6-0), and 8 wpi (early senescent; S8-0), as well as in S8 nodules that were treated for 2 weeks with 100 μM NaHS (S8-100). (B) Nitrogenase activity was measured in nodules at 6, 8, and 10 wpi (late senescent; S10-0), as well as in S10 nodules that were treated for 4 weeks with 100 μM NaHS (S10-100). ARA was measured in five biological replicates and each replicate contained nodules from five plants. ARA is expressed per nodule fresh weight. Means denoted by the same letter are not significantly different according to Duncan’s multiple range test (*P*<0.05).

The above results indicate that H_2_S and persulfidation protect protein function from overoxidation. To test the *in vivo* relevance of these findings, we investigated the effect of NaHS treatment on nodule functioning during natural senescence using *L. japonicus* plants with nodules at four different developmental stages: ‘young’, ‘mature’, ‘early senescent’, and ‘late senescent’, which correspond to 4, 6, 8, and 10 wpi, respectively. We chose to measure nitrogenase activity (ARA) because it integrates functioning of nodule host cells and bacteroids and is highly sensitive to nodule senescence. Two types of experiments were performed. First, we compared young, mature, and early senescent nodules. Nitrogenase activity was reduced by 51% and 60% in mature and early senescent nodules, respectively, compared with young nodules. Notably, treatment of plants with 100 μM NaHS for 2 weeks had a clear beneficial effect on SNF because nitrogenase activity of early senescent nodules was 83% higher than the untreated nodules of the same age and it was only 27% lower than the activity of young nodules (Fig. 8A). To investigate if the protective effect of H_2_S extends to later stages of senescence, we performed a second experiment by comparing mature, early senescent, and late senescent nodules. Nitrogenase activity declined by 33% and 57%, respectively, in early senescent and late senescent nodules with respect to mature nodules. Again, we found that treatment of plants with 100 μM NaHS for 4 weeks delayed the decrease of SNF. Thus, in the presence of NaHS, the nitrogenase activity of 10 wpi nodules was similar to that of untreated nodules at 8 wpi (Fig. 8B).

## Discussion

Over the last decades, considerable progress has been made in understanding the functions of ROS and RNS in plant biology (Noctor and Foyer, 2016; Umbreen *et al.*, 2018; Castro *et al.*, 2021) and, in the case of legumes, in SNF (Puppo *et al*., 2005; Bruand and Meilhoc, 2019; Minguillón *et al.*, 2022). Recently, H_2_S has emerged as an important signaling molecule in plants and animals (Hancock, 2019; T. Chen *et al.*, 2020). In legumes, H_2_S enhances nodulation and SNF (Zou *et al.*, 2019) although nothing is known about the mechanisms involved. Because H_2_S-mediated protein persulfidation is a possible way by which H_2_S and other RSS regulate nodule physiology, we investigated the persulfidome of common bean nodules at different developmental stages. In animals, higher persulfidation levels have been associated to resistance to oxidative stress and extended life span (Zivanovic *et al.*, 2019). However, virtually nothing is known about the link between H_2_S/persulfidation and nodule senescence and their effects on protein function.

Here, we show that persulfidation is a common PTM in plant and bacterial proteins, pointing to an important role of H_2_S in intracellular signaling in both symbionts. Our results indicate that, in the plant fraction of legume nodules, persulfidation is involved in the regulation of core carbohydrate metabolism, ATP and reducing power generation, protein biosynthesis and homeostasis, and amino acid and nucleotide metabolism (Fig. 1A, B). Because obvious differences exist between leaf and nodule physiology, it is surprising that our results bear some resemblance to those obtained in Arabidopsis leaves (Aroca *et al.*, 2017). These authors showed significant over-representation of persulfidated proteins in processes such as carbon metabolism and glycolysis, translation, jasmonic acid biosynthesis, and abiotic stress responses. The presence of H_2_S may have also the capacity to modulate H_2_O_2_ and NO signaling and, indeed, our proteomic analysis shows that ~30% of the identified persulfidated plant proteins in nodules have homologs in Arabidopsis that are susceptible to sulfenylation or *S*-nitrosylation (Hu *et al.*, 2015; Huang *et al.*, 2019). The percentage increases up to 59% when the comparison is made with sulfenylated proteins of *Medicago truncatula* nodules (Oger *et al.*, 2012). These results suggest a complex crosstalk between H_2_S and ROS/RNS. In addition, many bacterial proteins involved in key metabolic processes including SNF were found to be persulfidated. However, the relevance and regulatory functions of persulfidation in bacteria remain largely unknown. High levels of NO and ROS inhibit nitrogenase activity (Becana *et al.*, 2000; Bruand and Meilhoc, 2019). On the contrary, a positive effect of H_2_S on nodulation and SNF has been reported (Zou *et al.*, 2019). Other studies, however, showed that RSS such as polysulfides significantly decreased nitrogenase activity (Fukudome *et al.*, 2020), suggesting that a strict regulation of H_2_S homeostasis is required in the nodules for optimal functioning.

Nodule senescence is a poorly understood developmental stage regulated by the complex interaction of multiple signals including ROS, RNS, antioxidants, and hormones (Puppo *et al.*, 2005; Matamoros *et al.*, 2013; Bruand and Meilhoc, 2019). We show that, in general, nodule aging is associated with a decrease in the persulfidation levels of plant proteins (Figs 2, 3). These results are consistent with the recent finding that persulfidation in animal cells decreases with advancing age (Zivanovic *et al.*, 2019). Because H_2_S and persulfidation may protect Cys residues from overoxidation and irreversible protein damage (Filipovic *et al.*, 2018), decreased levels of this PTM could shift the cellular redox status to more oxidizing conditions and thereby trigger nodule senescence. This explanation is supported by our previous work showing that aging of common bean nodules is linked to a decrease in antioxidant metabolites and an increase in oxidized proteins (Loscos *et al.*, 2008; Matamoros *et al.*, 2013). Of note, persulfidation levels of bacteroids were higher in senescent than in young nodules, suggesting that the nodule host cells undergo senescence prior to the bacteroids. However, this was not the case for nitrogenase, which showed greater persulfidation in young and mature nodules than in senescent nodules (Fig. 4). Furthermore, H_2_S delayed the decrease of SNF observed in aging nodules. The nitrogenase enzyme complex is very sensitive to autoxidation (Becana *et al.*, 2010) and, therefore, an adequate persulfidation level could avoid overoxidation and contribute to maintaining a higher activity during nodule senescence. Moreover, the interaction of H_2_S with other enzymes and signaling pathways is likely and might contribute also to the higher levels of nitrogenase activity observed in NaHS-treated nodules compared with the corresponding untreated nodules.

The hypothesis that H_2_S and persulfidation protect critical Cys residues of proteins from overoxidation and irreversible damage was tested using nodule soluble extracts and recombinant proteins. Our results suggest that, in common bean nodules, H_2_S at strictly regulated concentrations has a positive effect on the ascorbate–GSH cycle because it partially prevents the overoxidation of MR and DR, keeping them active under conditions of excess ROS. The H_2_S-mediated improvement of plant tolerance to various abiotic stresses has been established (reviewed by T. Chen *et al.*, 2020). For example, in pea (*Pisum sativum*) seedlings, the supply of NaHS increased the content of H_2_S and protected the ascorbate–GSH enzyme activities from the inhibition caused by arsenate, reducing the contents of ROS and oxidized lipids and proteins (Singh *et al.*, 2015). Similarly, in wheat (*Triticum aestivum*) leaves under water stress, pretreatment with NaHS increased the activities of APX, GR, and DR and the contents of total and reduced ascorbate and glutathione, and decreased malondialdehyde level and electrolyte leakage (Shan *et al.*, 2011). In soybean, H_2_S alleviated the drought-induced decrease in nitrogenase activity, strengthened the plant antioxidant capacity, and reduced the oxidative damage (Lin *et al.*, 2022).

Previous studies in our laboratory showed that cytosolic Gpx3 of *L. japonicus* fulfills a protective function against oxidative stress, is involved in ROS and NO signaling, and is highly expressed in the infected zone of nodules (Matamoros *et al.*, 2015). Our experiments with the recombinant protein showed that under oxidative conditions the presence of a H_2_S donor partially maintains the protein in reduced form, probably through the production of persulfides. Because persulfides have more reactivity than the parent thiols, this PTM might cause an overall protein activation under oxidative conditions or changes in the protein redox state that could be relevant in redox signaling and in the protection of aging nodules against oxidative stress. Similarly, our finding of persulfidated Glb3-1 *in vivo* is relevant and, although the mechanism for the reaction of Glb3-1 with H_2_S is beyond the scope of this study, a role of H_2_S on the regulation of the biological activity of LjGlb3-1 can be proposed.

Hydrogen sulfide and persulfidation may also alter signaling pathways by interfering with Tyr nitration. This PTM was considered irreversible but denitration mechanisms have been described in animals that might exist also in plants (Kolbert *et al.*, 2017). The relevance of protein nitration in redox signaling is still not understood but there is evidence that it interferes with the phosphorylation of the Tyr residue. Moreover, Tyr nitration has been associated to nitro-oxidative damage in plants and animals and usually impairs protein function (Corpas *et al.*, 2013; Kolbert *et al.*, 2017; Bartesaghi and Radi, 2018). Here, we show that H_2_S/persulfidation decreases Tyr nitration levels of three recombinant proteins involved in ROS and NO homeostasis. Whereas a protective role can be anticipated, detailed case study approaches will be necessary to establish how persulfidation of individual proteins regulates their function and thereby plant redox homeostasis.

In summary, this study shows that protein persulfidation is a common PTM in legume nodules. As occurs in animal cells, persulfidation levels dropped abruptly in aging nodules and this may be related to the senescence process. Our results clearly point to a protective role of H_2_S and persulfidation on SNF and enzymes and proteins especially sensitive to oxidation by ROS and/or RNS. Moreover, H_2_S seems to modulate ROS- and RNS-mediated signaling through the regulation, via persulfidation, of the function and stability of proteins involved in H_2_O_2_ and NO metabolism. Understanding these processes will improve also our knowledge on the mechanisms underlying plant and nodule senescence in response to environmental cues.

## Supplementary data

The following supplementary data are available at *JXB* online.

Fig. S1. Singular enrichment analysis of genes coding for plant persulfidated proteins.

Fig. S2. Alignment of amino acid sequences of class 3 hemoglobins of *Lotus japonicus* and *Phaseolus vulgaris*.

Fig. S3. Mass spectrometry analysis of *Lotus japonicus* recombinant hemoglobin LjGlb3-1 (Lj1g3v2035270).

Fig. S4. Mass spectrometry analysis of *Lotus japonicus* recombinant glutathione peroxidase LjGpx3 (LotjaGi4g1v0458000).

Fig. S5. Predicted nitration sites according to iNitro-Tyr (app.aporc.org/iNitro-Tyr/).

Table S1. Plant proteins that were found to be persulfidated only at the ‘young’ developmental stage of nodules.

Table S2. Plant proteins that showed significant higher levels of persulfidation in ‘young’ than in ‘mature’ nodules.

Table S3. Plant proteins that were found to be persulfidated only at the ‘mature’ developmental stage of nodules.

Table S4. Plant proteins that showed significant higher levels of persulfidation in ‘mature’ than in ‘young’ nodules.

Table S5. Plant proteins that showed significant higher levels of persulfidation in ‘mature’ than in ‘senescent’ nodules.

Table S6. Plant proteins that were found to be persulfidated only at the ‘senescent’ developmental stage of nodules.

Table S7. Plant proteins that showed significant higher levels of persulfidation in ‘senescent’ than in ‘mature’ nodules.

Table S8. Bacterial proteins that showed significant higher levels of persulfidation in ‘young’ and ‘mature’ than in ‘senescent’ nodules.

Table S9. Bacterial proteins that showed significant higher levels of persulfidation in ‘senescent’ than in ‘young’ and ‘mature’ nodules.

Dataset S1. Proteins identified (FDR<1%) in ‘young’ nodules.

Dataset S2. Proteins identified (FDR<1%) in ‘mature’ nodules.

Dataset S3. Proteins identified (FDR<1%) in ‘senescent’ nodules.

erad436_suppl_Supplementary_Dataset_S1

erad436_suppl_Supplementary_Dataset_S2

erad436_suppl_Supplementary_Dataset_S3

erad436_suppl_Supplementary_Figures_S1-S5

erad436_suppl_Supplementary_Tables_S1-S9

## Data Availability

The data that support the findings of this study are available from the corresponding author upon reasonable request. The mass spectrometry proteomics data have been deposited in the ProteomeXchange Consortium via the PRIDE (Perez-Riverol *et al.*, 2022) partner repository with identifier PXD039852.
