# Supplementary material for: Persulfidation of plant and bacteroid proteins is involved in legume nodule development and senescence
Source: J Exp Bot. 2023 Nov 11;75(10):3009–25. doi: 10.1093/jxb/erad436 (PMC11103110; doi:10.1093/jxb/erad436)
Supplement: erad436_suppl_Supplementary_Figures_S1-S5 [file erad436_suppl_supplementary_figures_s1-s5.pdf]

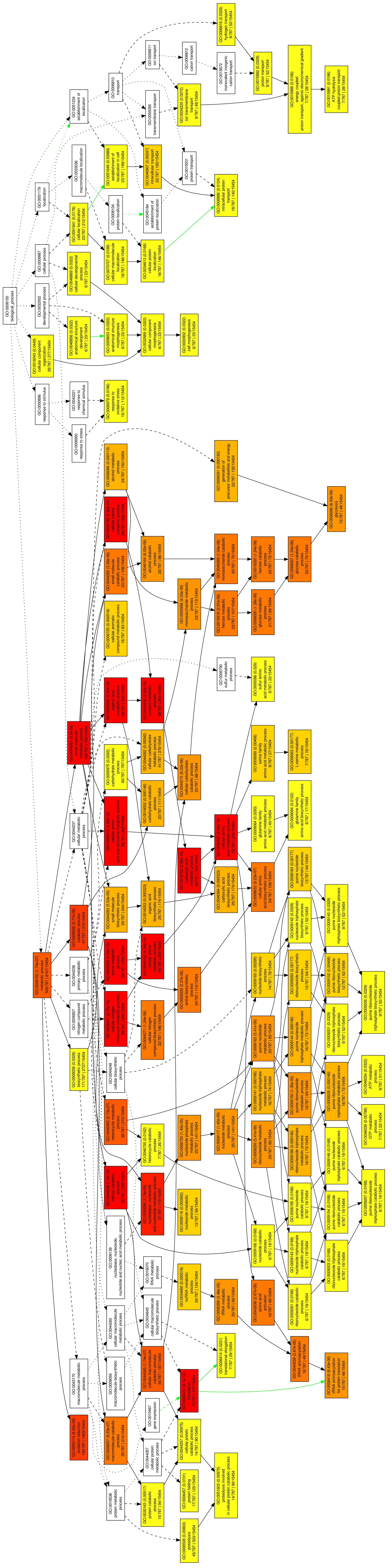

Supplementary Figure S1. Singular enrichment analysis (SEA) of genes coding for proteins that are persulfidated *in vivo* in biological process GO categories according to AgriGO v2.0 tool (http://systemsbiology.cau.edu.cn/agriGOv2/index.php).

```
AtGlb3          MQSLQDKASVLSGVDQAEAFAIDESNLFDKLGLQTFINLSTNFYTRVYDDEEEWFQSIFS   60
LjGlb3-1        MQSLQHKASEWSGVLTSNAFAIDDTNLFEKLGLQTFISLSTNFYNRVYDDEEEWFHLIFA   60
PvGlb3-1        MQSLQQKASEWSGISIDDAFAIDDADLFHTLGFQTFVTLSTNFYNRVYDDDEEWFRSIFG   60
                *****.***  **:   :*****:::**..**:***:.******.*****:****: **.

AtGlb3          NSNKEDAIQNQYEFFVQRMGGPPLYSQRKGHPALIGRHRPFPVTHQAAERWLEHMQNALD   120
LjGlb3-1        NSDKQIAIQNQYEFLVQRMGGPPLYSQRRGHPALIARHRAFPVTHEAAERWLHHMQQAVD   120
PvGlb3-1        NSEKEKAIQNQYEFLVQRMGGPPLYSQRRGHPALIARHRPFPLTHEAAERWLHHMAQALE   120
                **:*: ********:*************:******.*** **:**:******.** :*::

AtGlb3          DSVDIDQDSKIKMMKFFRHTAFFLVAGNELKNQNEKPKHKPQCACKHAANKPAEE-        175
LjGlb3-1        TSSDIDDDSKIKLMNFFRHTAYFIVAGIELKNQNFQM------PCKNA-PSPCKNF        169
PvGlb3-1        STSAIDDDSKIKLTNFFRHTAYFLVAGVEVKDQNHHT------PCKDAAQHPCKNF        170
                 :  **:*****: :******:*:*** *:*:** :        **.*   *.::*
```

Supplementary Figure S2. Amino acid sequences of class 3 phytoglobins (Glbs) from *Arabidopsis thaliana*, *Lotus japonicus*, and *Phaseolus vulgaris*. Residues in blue lettering are identical in the three species. Residues in yellow lettering are identical in the two legume species. In red, the highly conserved cysteine residue at the C-terminal of class 3 Glbs. Phytozome (https://phytozome-next.jgi.doe.gov/) identifiers: AtGlb3 (AT4G32690), LjGlb3-1 (Lj1g0018927.1), PvGlb3-1 (Phvul.009G104900.1).

A

1 MQSLQHKASE WSGVLTSNAF AIDDTNLFEK LGLQTFISLS TNFYNRVYDD
51 EEEWFHLIFA NSDKQIAIQN QYEFLVQRMG GPPLYSQRRG HPALIARHRA
101 FPVTHEAAER WLHHMQQAVD TSSDIDDDSK IKLMNFFRHT AYFIVAGIEL
151 KNQNFQMPCK NAPSPCKN

B

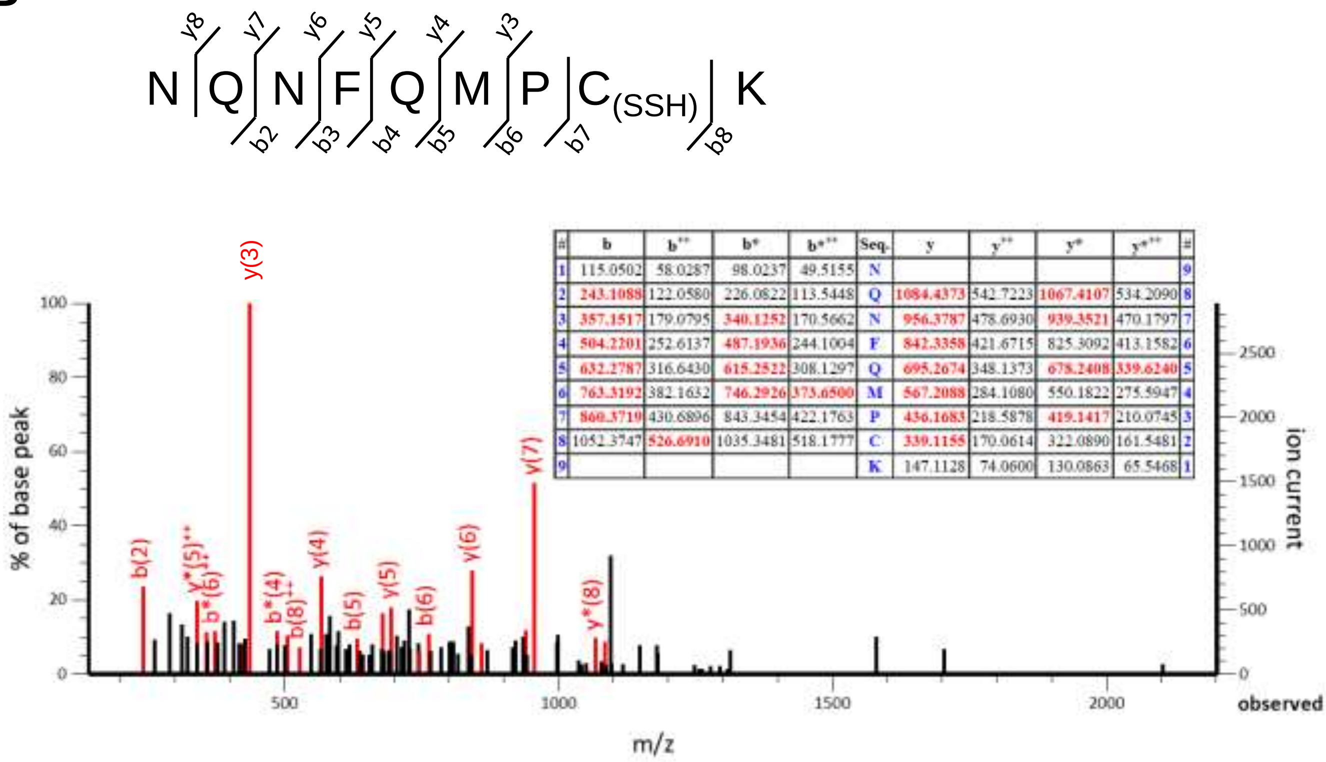

#	b	b^{++}	b^{*}	b^{*++}	Seq.	y	y^{++}	y^{*}	y^{*++}	#
1	115.0502	58.0287	98.0237	49.5155	N					9
2	243.1088	122.0580	226.0822	113.5448	Q	1084.4373	542.7223	1067.4107	534.2090	8
3	357.1517	179.0795	340.1252	170.5662	N	956.3787	478.6930	939.3521	470.1797	7
4	504.2201	252.6137	487.1936	244.1004	F	842.3358	421.6715	825.3092	413.1582	6
5	632.2787	316.6430	615.2522	308.1297	Q	695.2674	348.1373	678.2408	339.6240	5
6	763.3192	382.1632	746.2926	373.6500	M	567.2088	284.1080	550.1822	275.5947	4
7	860.3719	430.6896	843.3454	422.1763	P	436.1683	218.5878	419.1417	210.0745	3
8	1052.3747	526.6910	1035.3481	518.1777	C	339.1155	170.0614	322.0890	161.5481	2
9					K	147.1128	74.0600	130.0863	65.5468	1

Supplementary Figure S3. Analysis of *Lotus japonicus* recombinant phytoglobin LjGlb3-1 (Lj1g3v2035270.1) using mass spectrometry. A, The protein was identified with a sequence coverage of 89%; the identified peptides are shown in red, and the peptide containing persulfidated Cys[159] is shown underlined. B, MS/MS fragmentation spectra of the tryptic peptide containing Cys[159] of LjGlb3-1. The table inside the spectrum contains the predicted ion types for the modified peptide, and the ions detected in the spectrum are highlighted in red color.

A

1 MQLLTFWNWI SLVILAFAFF FFFFFFYSQT HPASPPSPST MAEQTSKSLY
51 DFTVKDIRGN DVSLSQYSGK VLIIVNVASQ CGLTQTNYKE LNILYEKYKS
101 KGLEILAFPC NQFAGQEPGT NDEIQDVVCT RFKSEFPVFD KVEVNGKNAE
151 PLFKFLKDQK GGIFGDGIKW NFTKFLVNKE GKVVERYAPT TSPMKIEKDL
201 EKLLQSS

B

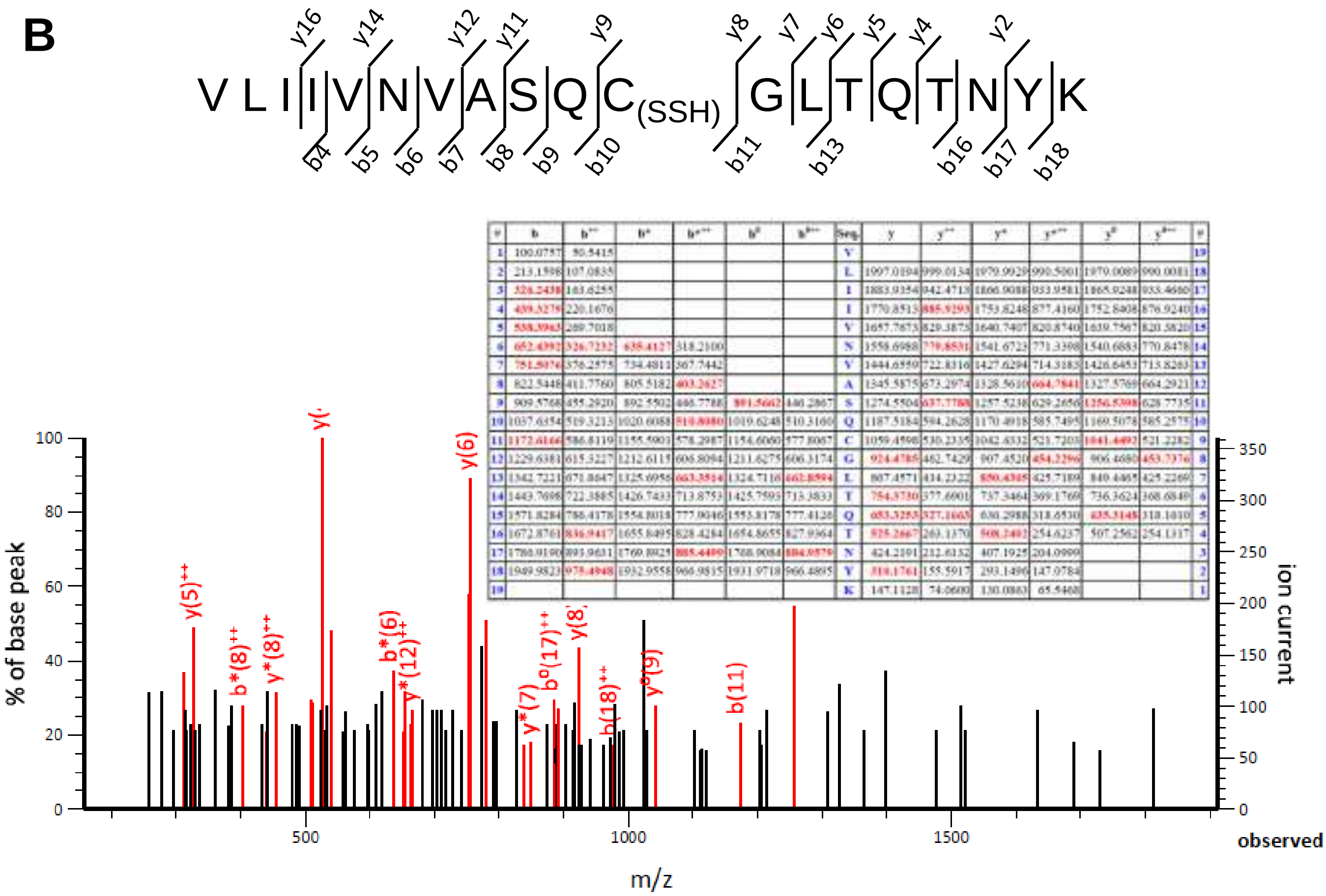

#	b	b++	b*	b*++	b°	b°++	Seq.	y	y++	y*	y*++	y°	y°++	#
1	100.0757	50.5415					V							19
2	213.1598	107.0835					L	1997.0194	999.0134	1979.9929	990.5001	1979.0089	990.0081	18
3	326.2438	163.6255					I	1883.9354	942.4713	1866.9088	933.9581	1865.9248	933.4660	17
4	439.3279	220.1676					I	1770.8513	885.9293	1753.8248	877.4160	1752.8408	876.9240	16
5	538.3963	269.7018					V	1657.7673	829.3873	1640.7407	820.8740	1639.7567	820.3820	15
6	652.4392	326.7232	635.4127	318.2100			N	1558.6988	779.8531	1541.6723	771.3398	1540.6883	770.8478	14
7	751.5076	376.2575	734.4811	367.7442			V	1444.6559	722.8316	1427.6294	714.3183	1426.6453	713.8263	13
8	822.5448	411.7760	805.5182	403.2627			A	1345.5875	673.2974	1328.5610	664.7841	1327.5769	664.2921	12
9	909.5768	455.2920	892.5502	446.7788	891.5662	446.2867	S	1274.5504	637.7788	1257.5238	629.2656	1256.5398	628.7735	11
10	1037.6354	519.3213	1020.6088	510.8080	1019.6248	510.3160	Q	1187.5184	594.2628	1170.4918	585.7495	1169.5078	585.2575	10
11	1172.6166	586.8119	1155.5901	578.2987	1154.6060	577.8067	C	1059.4598	530.2335	1042.4332	521.7203	1041.4492	521.2282	9
12	1229.6381	615.3227	1212.6115	606.8094	1211.6275	606.3174	G	924.4785	462.7429	907.4520	454.2296	906.4680	453.7376	8
13	1342.7221	671.8647	1325.6956	663.3514	1324.7116	662.8594	L	867.4571	434.2322	850.4305	425.7189	849.4465	425.2269	7
14	1443.7698	722.3885	1426.7433	713.8753	1425.7593	713.3833	T	754.3730	377.6901	737.3464	369.1769	736.3624	368.6849	6
15	1571.8284	786.4178	1554.8018	777.9046	1553.8178	777.4126	Q	653.3253	327.1663	636.2988	318.6530	635.3148	318.1610	5
16	1672.8761	836.9417	1655.8495	828.4284	1654.8655	827.9364	T	525.2667	263.1370	508.2402	254.6237	507.2562	254.1317	4
17	1786.9190	893.9631	1769.8925	885.4499	1768.9084	884.9579	N	424.2191	212.6132	407.1925	204.0999			3
18	1949.9823	975.4948	1932.9558	966.9815	1931.9718	966.4895	Y	310.1761	155.5917	293.1496	147.0784			2
19							K	147.1128	74.0600	130.0863	65.5468			1

Supplementary Figure S4. Analysis of *Lotus japonicus* recombinant glutathione peroxidase LjGpx3 (LotjaGi4g1v0458000) using mass spectrometry. A, The protein was identified with a sequence coverage of 74%; the identified peptides are shown in red, and the peptide containing persulfidated Cys[81] is shown underlined. B, MS/MS fragmentation spectra of the tryptic peptide containing Cys[81] of LjGpx3. The table inside the spectrum contains the predicted ion types for the modified peptide, and the ions detected in the spectrum are highlighted in red color.

Iron superoxide dismutase

```
>VuFeSOD
MASLGLQNVAGINLLFKEGPKVNAKFELKPPPYPLNGLEPVMSQQTLEFHWGKHHRTYVENLKKQVVGTE
LDGKSLEEIIVTAYNKGDILPAFNNAAQVWNHDFFWECMKPGGGGKPSGELLELIERDFGSFEKFLDEFK
AAAATQFGSGWAWLAYKASKLDGENAANPPSADEDNKLVVIKSPNAVNPLVWGGYYPLLTIDVWEHAYYL
DFQNRRPDYISVFMDKLVSWDAVSSRLEQAKALSA
```

```
>PvFeSOD_
MASLGGLQNVTGINLLFKEGPKVNAKFELKPPPYPLNGLEPVMSQQTLEFHWGKHHKTYVENLKKQVVGT
ELDGKSLEEIIVTSYNKGDILPAFNNAAQVWNHDFFWECMKPGGGGKPSGELLELIERDFGSFEKFLDEF
KAAAATQFGSGWAWLAYRASKLGGENAENPPSTHEDNKLVVIKSPNAVNPLVWGSYYPLLTIDVWEHAYY
LDFQNRRPDYISVFMDKLVSWDAVSSRLEQAKAF
```

Glutathione peroxidase 3

```
>LjGpx3
MAEQTSKSLYDFTVKDIRGNDVSLSQYSGKVLIIVNVASQCGLTQTNYKELNILYEKYKSKGLEILAFPC
NQFAGQEPGTNDEIQDVVCTRFKSEFPVFDKVEVNGKNAEPLFKFLKDQKGGIFGDGIKWNFTKFLVNKE
GKVVERYAPTTSPMKIEKDLEKLLQSS
```

```
>PvGpx3

MAEESSKSIYDFTVKDIRGNDVSLNDFTGKVILIVNVASQCGLTQTNYKELNVLYDKYKNQGFEILAFPC
NQFAGQEPGNNEEIQDVVCTRFKAEFPIFDKVEVNGKNAAPLYKFLKEQKGGIFGDGIKWNFTKFLVNKE
GKVVERYAPTTSPLKIEKDIEKLLQS
```

Class 3 phytoglobin

```
>LjGlb3-1
MQSLQHKASEWSGVLTSNAFAIDDTNLFEKLGLQTFISLSTNFYNRVYDDEEEWFHLIFANSDKQIAIQN
QYEFLVQRMGGPPLYSQRRGHPALIARHRAFPVTHEAAERWLHHMQQAVDTSSDIDDDSKIKLMNFFRHT
AYFIVAGIELKNQNFQMPCKNAPSPCKN
```

```
>PvGlb3-1
MQSLQQKASEWSGISIDDAFAIDDADLFHTLGFQTFVTLSTNFYNRVYDDDEEWFRSIFGNSEKEKAIQN
QYEFLVQRMGGPPLYSQRRGHPALIARHRPFPLTHEAAERWLHHMAQALESTSAIDDDSKIKLTNFFRHT
AYFLVAGVEVKDQNHHTPCKDAAQHPCKNF
```

Supplementary Figure S5. Predicted nitrotyrosine sites (highlighted in blue) in cowpea (*Vigna unguiculata*) iron superoxide dismutase (VuFeSOD; GenBank: AAF28773.1), common bean (*Phaseolus vulgaris*) FeSOD (PvFeSOD; Phytozome: Phvul.007G135400), *Lotus japonicus* glutathione peroxidase 3 (LjGpx3; Lotus Base: LotjaGi4g1v0458000), common bean Gpx3 (PvGpx3; Phytozome: Phvul.002G322400), *L. japonicus* class 3 phytoglobin (LjGlb3-1; Lotus Base: Lj1g3v2035270.1), and common bean Glb3-1 (PvGlb3-1; Phytozome: Phvul.009G104900) according to iNitro-Tyr (app.aporc.org/iNitro-Tyr/).
